# Emergent Percutaneous Transluminal Coronary Angioplasty of an Occluded Giant Ectatic Coronary Aneurysm

**DOI:** 10.1016/j.jscai.2023.101039

**Published:** 2023-05-19

**Authors:** Jay M. Patel, Carlos Sisniega, Aleesha Shaik, Olcay Aksoy, Daniel Levi, Rushi V. Parikh

**Affiliations:** a Division of Cardiology, University of California, Los Angeles, Los Angeles, California; b Division of Pediatric Cardiology, University of California, Los Angeles, Mattel Children’s Hospital, Los Angeles, California

**Keywords:** giant coronary aneurysm, multisystem inflammatory syndrome in children

The incidence of giant coronary aneurysms (GCAs) and subsequent intracoronary thrombosis is approximately 0.02%, with most occurring due to Kawasaki disease or Takayasu arteritis.^1^ Currently, there are only a few reported cases of GCA secondary to multisystem inflammatory syndrome in children.^2^

This case report involves a 14-year-old boy with multisystem inflammatory syndrome in children secondary to COVID-19 infection in 2020, which was complicated by the development of GCAs of the entire coronary tree. In addition to immediate treatment with intravenous immunoglobulin and steroids, he was prescribed rivaroxaban and aspirin for his GCAs. He was in his usual state of health until he recently presented to a nearby emergency department with several hours of substernal chest pain that awoke him from sleep and radiated to his neck, jaw, and left arm. Examination was notable for stable hemodynamics. Serial electrocardiograms showed dynamic and progressive anterolateral ST elevations, and the initial troponin I concentration was 0.5 ng/mL. An urgent coronary computed tomography angiogram (CCTA) revealed serial GCAs of the right coronary artery and left circumflex artery and an occluded GCA of the proximal left anterior descending (LAD) coronary artery (Figure 1A, C). GCAs measured ≤12.4 mm in the right coronary artery, 13.5 mm in the left circumflex artery, and 9.6 mm in the LAD.

**Figure 1:**
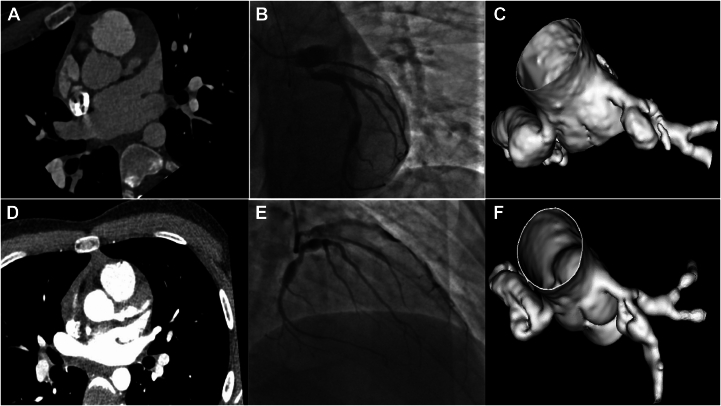
**LAD before intervention.** CCTA images (**A**) and 3D reconstruction (**C**) showed proximal occlusion of aneurysmal LAD, which was corroborated on angiography (**B**). Postangioplasty angiography demonstrated restoration of flow to the distal LAD (**E**). CCTA images (**D**) and 3D reconstruction (**F**) 48 hours later showed continued vessel patency. CCTA, coronary computed tomography angiogram; LAD, left anterior descending coronary artery

Given the acute myocardial infarction in the setting of a thrombosed proximal LAD GCA, the patient was emergently transferred to our institution where coronary angiography confirmed the findings on CCTA (Figure 1B; Supplemental Videos 1 and 2). We successfully crossed the organized thrombus using a Fielder XT coronary guide wire within a microcatheter. We deferred aspiration thrombectomy given the massive size of the thrombus and consequently low likelihood of efficacy. Rather, we performed serial percutaneous transluminal coronary angioplasty (using ≤3.0-mm semicompliant balloon), which resulted in restoration of TIMI grade II flow to the distal vessel (Figure 1E and Supplemental Video 3) and resolution of the patient’s symptoms. We elected against stent placement for GCAs because it would have resulted in severe malapposition. Finally, owing to extensive residual thrombus burden, we chose to prescribe the patient a robust antiplatelet/anticoagulant regimen of aspirin, ticagrelor, intravenous heparin (48 hours), and eptifibitide (24 hours).

The patient remained chest pain free, and the troponin I level peaked at 100 ng/mL. Repeat CCTA 48 hours later showed a patent LAD with a good distal flow (Figures 1D, F; Supplemental Video 4). A transthoracic echocardiogram demonstrated hypokinesis in the LAD territory and a left ventricular ejection fraction of 40% (previously at a normal range). He was discharged on short-term triple therapy (aspirin, ticagrelor, and warfarin) with planned discontinuation of aspirin at 1 month. At the 3-month follow-up, the patient reported no further chest pain.

## Supplementary material

Supplemental Video 1 Cine angiography of the RCA in a posteroanterior cranial projection revealed serial GCAs. GCA, giant coronary aneurysm; RCA, right coronary artery.

Supplemental Video 2 Cine angiography of the LCA in a RAO caudal projection showed an occluded proximal GCA of the LAD without distal flow. The LCx showed relatively smaller GCAs. GCA, giant coronary aneurysm; LAD, LAD, left anterior descending coronary artery; LCA, left coronary artery; LCx, left circumflex artery; RAO, right anterior oblique.

Supplemental Video 3 Cine angiography of the LCA in a RAO cranial projection demonstrated restoration of flow to the distal LAD with a significant residual thrombus burden. LAD, LAD, left anterior descending coronary artery; LCA, left coronary artery; RAO, right anterior oblique.

Supplemental Video 4 3D reconstruction model of the LAD GCA using CT angiography data before (top left) and after (bottom right) percutaneous transluminal coronary angioplasty. Postangioplasty model showed increased lumen extension of the LAD. GCA, giant coronary aneurysm; LAD, left anterior descending coronary artery
